# The P2X_7_ receptor tracer [^11^C]SMW139 as an *in vivo* marker of neuroinflammation in multiple sclerosis: a first-in man study

**DOI:** 10.1007/s00259-019-04550-x

**Published:** 2019-11-08

**Authors:** Marloes H. J. Hagens, Sandeep S. V. Golla, Bieneke Janssen, Danielle J. Vugts, Wissam Beaino, Albert D. Windhorst, James O’Brien-Brown, Michael Kassiou, Robert C. Schuit, Lothar A. Schwarte, Helga E. de Vries, Joep Killestein, Frederik Barkhof, Bart N. M. van Berckel, Adriaan A. Lammertsma

**Affiliations:** 1 MS Center Amsterdam, Amsterdam Neuroscience, Amsterdam UMC - location VUmc, De Boelelaan 1117, 1081 HV Amsterdam, The Netherlands; 2 Department of Neurology, Amsterdam Neuroscience, Amsterdam UMC - location VUmc, De Boelelaan 1117, 1081 HV, Amsterdam, The Netherlands; 3 Department of Radiology and Nuclear Medicine, Amsterdam Neuroscience, Amsterdam UMC - location VUmc, De Boelelaan 1117, 1081 HV Amsterdam, The Netherlands; 4 Department of Molecular Cell Biology and Immunology, Amsterdam UMC - location VUmc, De Boelelaan 1117, 1081 HV Amsterdam, The Netherlands; 5 Department of Anaesthesiology, Amsterdam UMC - location VUmc, De Boelelaan 1117, 1081 HV, Amsterdam, The Netherlands; 6 School of Chemistry, University of Sydney, Sydney, Australia; 7 Institutes of Neurology and Healthcare Engineering, UCL Institute of Neurology, London, UK

**Keywords:** Multiple sclerosis, Positron emission tomography, Neuroinflammation, [^11^C]SMW139, Purinergic signalling, P2X_7_-receptor

## Abstract

**Purpose:**

The novel PET tracer [^11^C]SMW139 binds with high affinity to the P2X_7_ receptor, which is expressed on pro-inflammatory microglia. The purposes of this first in-man study were to characterise pharmacokinetics of [^11^C]SMW139 in patients with active relapsing remitting multiple sclerosis (RRMS) and healthy controls (HC) and to evaluate its potential to identify *in vivo* neuroinflammation in RRMS.

**Methods:**

Five RRMS patients and 5 age-matched HC underwent 90-min dynamic [^11^C]SMW139 PET scans, with online continuous and manual arterial sampling to generate a metabolite-corrected arterial plasma input function. Tissue time activity curves were fitted to single- and two-tissue compartment models, and the model that provided the best fits was determined using the Akaike information criterion.

**Results:**

The optimal model for describing [^11^C]SMW139 kinetics in both RRMS and HC was a reversible two-tissue compartment model with blood volume parameter and with the dissociation rate k_4_ fixed to the whole-brain value. Exploratory group level comparisons demonstrated an increased volume of distribution (V_T_) and binding potential (BP_ND_) in RRMS compared with HC in normal appearing brain regions. BP_ND_ in MS lesions was decreased compared with non-lesional white matter, and a further decrease was observed in gadolinium-enhancing lesions. In contrast, increased V_T_ was observed in enhancing lesions, possibly resulting from disruption of the blood-brain barrier in active MS lesions. In addition, there was a high correlation between parameters obtained from 60- to 90-min datasets, although analyses using 60-min data led to a slight underestimation in regional V_T_ and BP_ND_ values.

**Conclusions:**

This first in-man study demonstrated that uptake of [^11^C]SMW139 can be quantified with PET using BP_ND_ as a measure for specific binding in healthy controls and RRMS patients. Additional studies are warranted for further clinical evaluation of this novel neuroinflammation tracer.

**Electronic supplementary material:**

The online version of this article (10.1007/s00259-019-04550-x) contains supplementary material, which is available to authorized users.

## Introduction

Radiological evaluation of relapsing remitting multiple sclerosis (RRMS) is mainly based on new T2 lesions and active gadolinium-enhancing lesions on magnetic resonance imaging (MRI) [1]. However, detection of these disease-specific lesions by MRI only partly demonstrates the underlying pathophysiological processes in MS. As positron emission tomography (PET) can be used to visualise distinct molecular processes *in vivo*, it may provide unique insights in the pathophysiology of neuroinflammation (and neurodegeneration) in MS [2]. Over the past decades, the translocator protein 18 kDa (TSPO), present on the mitochondrial membrane of microglial cells, has been used as an *in vivo* marker of neuroinflammation in several neurological disorders including MS [3–5]. Although in general results have been positive, the use of TSPO as a marker for neuroinflammation has some limitations, such as an intracellular binding site, genetic polymorphisms, and additional binding sites on monocytes and vascular wall endothelium [6, 7]. In addition, TSPO does not differentiate between resting state and pro-inflammatory and neuro-protective microglia subtypes [8]. Therefore, new PET tracers are needed, which specifically target proteins that reflect the status of microglial cells. Recently, using well-characterised post-mortem tissues of patients with MS and activated human microglia, it has been demonstrated that the purinergic P2X_7_ receptor is highly expressed on pro-inflammatory microglia and macrophages, is selectively expressed within MS lesions, and may be involved in the neuroinflammatory cascade [8–10]. To a lesser extent, expression of P2X_7_ receptor has also been found in grey matter on astrocytes, oligodendrocytes, and neurons [11]. Moreover, it was shown that the radioligand [^11^C]SMW139 selectively binds to the P2X_7_ receptor with high affinity in preclinical *in vivo* studies and in post-mortem human tissues [8, 12–14].

However, so far, no human *in vivo* data are available for this tracer and its validity to visualise activated microglia and macrophages. The aims of this study were therefore to evaluate *in vivo* pharmacokinetic characteristics of [^11^C]SMW139 and to perform a proof of concept study assessing whether this novel tracer can be used for identifying neuroinflammation in RRMS.

## Material and methods

### Subjects

All subjects were recruited between June 2017 and April 2018 from the MS Center of the Amsterdam UMC, location VUmc. Patients were diagnosed with relapsing remitting MS according to the 2017 revisions of the McDonald criteria, and they had active disease at recruitment [15]. Healthy controls were age-matched to the patients. All subjects were screened for relevant neurological, immunological, cardiac, renal, and haematological diseases using medical history, physical and neurological examinations, and blood tests. Patients were not allowed to use immunomodulating medication at the time of the PET-scan, taking into account a washout of 2–4 weeks for first-line treatment, 6–12 weeks for second-line treatment, and 6 weeks for intravenous methylprednisolone.

This study was approved by the Medical Ethics Review Committee of the Amsterdam UMC, location VUmc (2016.548). Written informed consent was obtained from all subjects prior to the first study activity.

### MRI scanning protocol

MR imaging was performed on a 3-T General Electric Discovery MR750 system within 7 days of the PET scan. MR imaging included 3D T1 (repetition time 8.2 ms, echo time 3.2 ms, flip angle 12°, measured voxel size 1.0 × 1.0 × 1.0 mm^3^) for structural information. For patients, a 3D fluid attenuation inversion recovery (FLAIR) (repetition time 8000 ms, echo time 130 ms, inversion time 2340 ms, measured voxel size 1.1 × 1.1 × 1.2 mm^3^) and a post-contrast SE T1 (repetition time 660 ms, echo time 9 ms, measured voxel size 0.8 × 1.0 × 3.0 mm^3^) were used for segmentation of the gadolinium-enhancing and non-enhancing MS lesions.

### PET scanning protocol

Radiosynthesis of [^11^C]SMW139 was performed following the procedures described by Janssen et al., with slight modification since K_2_CO_3_ (5 mg) was used as a base instead of NaOH [12]. The tracer was obtained with a > 98% radiochemical purity. PET scans were performed on an Ingenuity TF PET-CT scanner (Philips Medical Systems, Best, The Netherlands). Following an automated intravenous infusion of a bolus of 362 ± 44 MBq [^11^C]SMW139 (molar activity of 59 ± 38 GBq μmol^−1^ at time of injection) a 90-min dynamic PET scan was acquired (see Online Resource 1 for information per subject). Emission data were collected in list mode and reconstructed into a dynamic dataset of 22 frames (1 × 15, 3 × 5, 3 × 10, 4 × 60, 2 × 150, 2 × 300, 7 × 600 s) using a standard reconstruction algorithm (BLOB-OS-TF) including corrections for scatter, randoms, attenuation, and dead time, and with a final voxel size of 2 × 2 × 2 mm^3^.

### Input functions

Automated continuous arterial blood sampling from a radial artery cannula was performed at a withdrawal rate of 5 mL min^−1^ for the first 5 min and 2.5 mL min^−1^ for the remainder of the scan. At set times (5, 10, 20, 40, 60, 75, and 90 min), continuous withdrawal was interrupted briefly for the collection of manual arterial blood samples [16]. Manual plasma samples were analysed using high-performance liquid chromatography (HPLC) to determine fractions of intact [^11^C]SMW139 and its radioactive metabolites, as has been described in more detail before [17]. In brief, plasma supernatant was separated from blood cells and diluted with 2 mL of water and loaded onto an activated tC18 Sep-Pak, followed by washing with 3 mL of water to obtain the polar fraction. The non-polar fraction was then eluted with 2 mL of MeOH and 1 mL of water and further analysed by HPLC. As a stationary phase, a Gemini C18 5-μm (10 × 250 mm) column with a gradient of acetonitrile (A) and 0.1% DIPA (B) as eluent was used. Metabolite-corrected arterial plasma input functions were obtained by correcting the arterial whole-blood TACs for both plasma to whole-blood ratios, metabolite fraction, and time delay.

### Image processing

For the MS patients, T2 lesions were segmented on the FLAIR scans using the automated segmentation tool *k*NN-TPP [18]. Next, lesion filling of the T1-weighted scans was performed using LEAP [19]. Next, the T1-weighted scans were registered to the summed PET images and this coregistration was then applied to the whole dynamic PET scan. Subsequently, segmentation of grey matter, white matter, and cerebrospinal fluid of the co-registered MRI scans was performed using SPM12 software [20]. PVElab [21] was used for region of interest (ROI) definition according to the Hammers template [22]. By superimposing these ROIs onto the dynamic PET scans, regional time activity curves (TACs) were generated. In addition, cortical grey matter ROIs were combined to define larger cortical ROIs, e.g., frontal cortex, temporal cortex, and cingulate cortex. Moreover, segmented T2 lesions were subtracted from the white matter mask to define non-lesional white matter. Finally, lesions with gadolinium enhancement on the post-contrast T1-weighted scans were identified on the segmented T2 lesion masks, to obtain a separate mask for enhancing lesions only.

### Kinetic modelling

To identify the optimal tracer kinetic model for describing [^11^C]SMW139 kinetics, standard single-tissue (1T2k) together with reversible and irreversible two-tissue (2T3k and 2T4k, respectively) compartment models was used, both with and without blood volume parameter (V_B_). In addition, a dual run 2T4k_V_B_ model was used in which the first run was used to estimate whole-brain grey and white matter k_4_, which subsequently was used to fix k_4_ for individual grey and white matter ROIs in the second run (2T4k_V_B__k_4_). This analysis was added to reduce the number of fit parameters, thereby improving precision of the other fit parameters. The Akaike information criterion (AIC) was used to compare fits of different models [23]. In addition, reliability of parameter estimates was evaluated using the percentage standard error (%SE) in parameter estimates. For more robust parameters, a %SE cut-off of 25% was used for K_1_, k_2_, and V_B_, and a cut-off of 50% for k_3_ and k_4_.

### Group differences

Differences in volume of distribution (V_T_) and binding potential (BP_ND_, defined as k_3_/k_4_) derived using the optimal model were used to compare the patient group with the healthy controls. Per subject, ROIs with %SE higher than the cut-off for the various parameters were excluded from this analysis. Exploratory evaluation of the group differences was carried out using an independent *t* test in SPSS 22.0 (IBM Corp., Armonk, NY).

### Scan duration

To evaluate the impact of a shorter scan duration on parameter estimates, analysis was repeated using only 60 min data. Comparisons were performed using linear regression and Bland-Altman analysis.

## Results

### Cohort description

Demographic information for the subjects included in this study is provided in Table 1, and additional information is available in Online Resource 1. The RRMS patients and healthy controls were comparable with respect to age and gender. All RRMS patients had a short disease duration, with a median of less than 1 year. All five patients had at least one clinical relapse in the 6 months prior to study participation, with one or more gadolinium-enhancing lesions on the clinical MRI. Three patients where still recovering from a recent relapse at the time of study participation. Four patients had at least one gadolinium-enhancing lesion on the study MRI.

**Table 1:** Cohort description

	RRMS (*n* = 5)	HC (*n* = 5)
Age, mean ± SD (years)	38.6 ± 12.5*	36.6 ± 13.5*
Gender, male/female	2/3	2/3
Disease duration, median and range (years)	0.8 (0.1–5.0)	
EDSS, median and range	3.0 (2.0–5.5)	
T25FW, median and range	4.4 (3.8–8.0)	
9-HPT, median and range	24.4 (16.9–25.7)	
SDMT, median and range	44 (33–57)	
T2 lesion volume, median and range (cm^3^)	18.1 (0.5–31.1)	
Volume gadolinium enhancing lesions, median and range (cm^3^)	0.7 (0–10.8)	

*EDSS* Expanded Disability Status Scale, *HC* healthy control, *RRMS* relapsing remitting multiple sclerosis

*Mann-Whitney *U* test: *p* = 0.84

### PET image

**Fig. 1:**
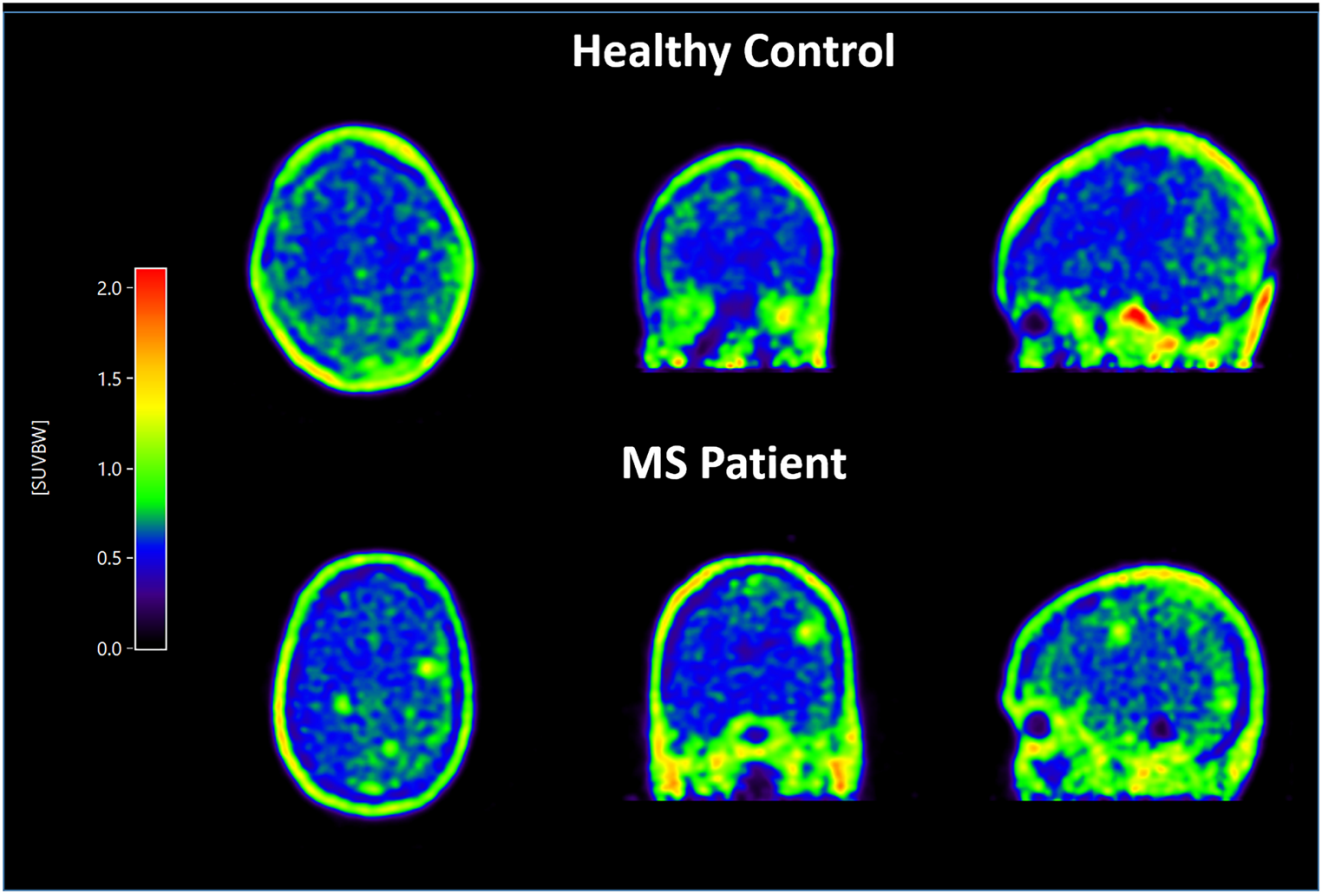
An SUV image of one healthy control and one patient

Figure 1 provides SUV images of one healthy control and one MS patient.

### Tracer kinetics

**Fig. 2:**
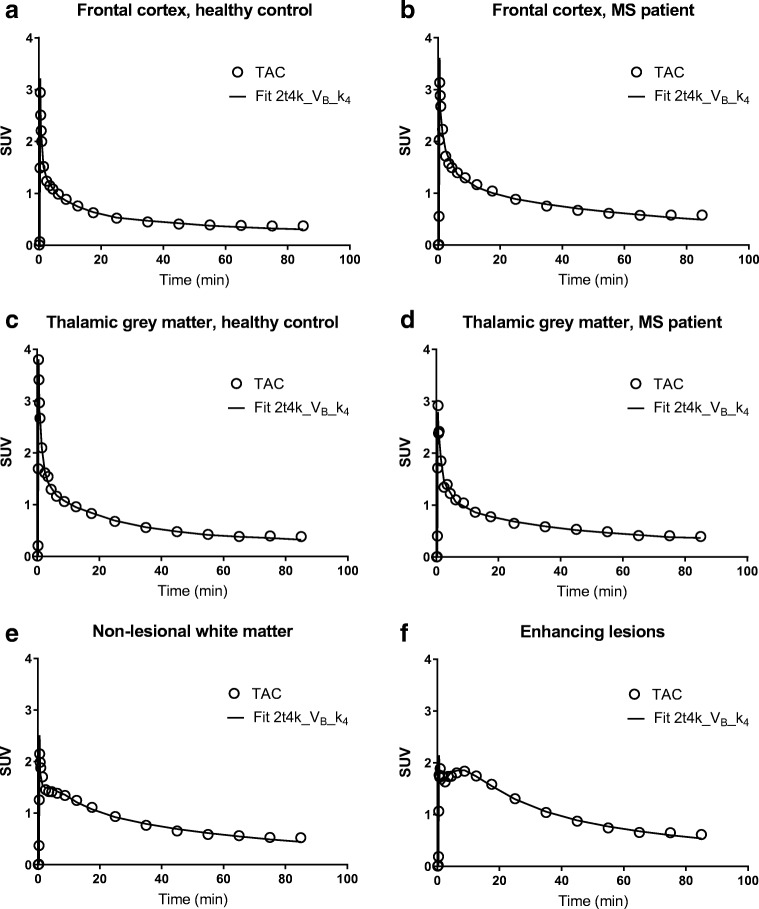
Model fits. Typical time activity curves (TACs) and corresponding fits obtained using the 2t4k_V_B__k_4_ model for the frontal cortex of a healthy control (**a**) and an RRMS patient (**b**), the thalamic grey matter for a healthy control (**c**) and an RRMS patient (**d**), and the non-lesional white matter (**e**) and MS lesions of an MS patient (**f**). Standardized uptake values (SUV) have been calculated as the tissue radioactivity concentration divided by the injected activity per kilogramme of body weight

Based on the AIC, pharmacokinetics of [^11^C]SMW139 were best described by the 2T4k_V_B__k_4_ model. This model was preferred in all subjects, and only in a few ROIs, 2T4k_V_B_ or 2T3k_V_B_ was preferred. Figure 2 illustrates a few model fits for the TACs of grey and white matter ROIs of representative patients and controls. These TACs demonstrate fast clearance of [^11^C]SMW139. Mean micro/macro parameters for all the grey and white matter regions of interest, estimated using the optimal model, are presented in Table 2. The rate of [^11^C]SMW139 entering the brain, which is expressed by K_1_, was comparable between patients and controls. However, the rate of efflux from the non-displaceable compartment to the blood, represented by k_2_, was high, being higher in controls than in patients. In addition, the rate of influx to the specific binding compartment, expressed by k_3_, was different between patients and controls, but small in both groups. This rapid clearance from the non-displaceable compartment combined with a low rate of specific binding resulted in a small specific compartment for [^11^C]SMW139. This made kinetic parameter estimation for this specific compartment prone to noise, resulting in unreliable k_4_ estimates in smaller ROIs (data not shown). Fixing k_4_ to within-subject whole-brain grey and white matter values (2T4k_V_B__k_4_ model) improved reliability of the model estimates. As Online Resource 2 shows, the %SE for 2T4k_V_B__k_4_ parameter estimates were low, except for very few ROIs.

**Table 2:** Mean parameter estimates 2T4k_V_B__k_4_ for whole-brain grey and white matter for all relapsing remitting multiple scleroses (RRMS) patients and healthy controls (HC)

	K_1_	k_2_	k_3_	k_4_	V_B_
Grey matter					
RRMS	0.087 ± 0.030	0.535 ± 0.115	0.035 ± 0.010	0.020 ± 0.008	0.075 ± 0.019
HC	0.085 ± 0.022	0.686 ± 0.127	0.032 ± 0.012	0.029 ± 0.009	0.075 ± 0.015
*p* value*	0.44	< 0.001	0.001	0.17	0.83
White matter					
RRMS	0.062 ± 0.025	0.378 ± 0.150	0.043 ± 0.017	0.032 ± 0.012	0.057 ± 0.016
HC	0.062 ± 0.020	0.526 ± 0.146	0.057 ± 0.021	0.058 ± 0.019	0.054 ± 0.012
*p* value*	0.88	< 0.001	< 0.001	0.041	0.061

*HC* healthy control, *RRMS* relapsing remitting multiple sclerosis

*Independent *t* test in SPSS 22.0

### Group differences

**Fig. 3:**
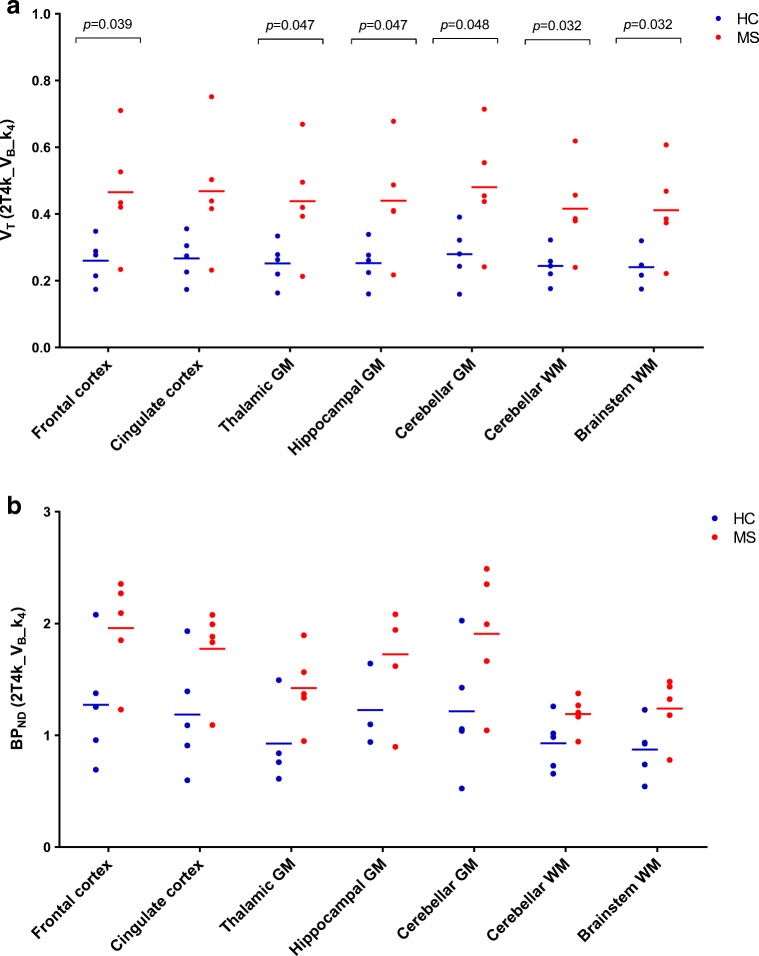
Regional volume of distribution and binding potential. **a** V_T_ and **b** BP_ND_ values of the five healthy controls (HC) in blue and five multiple sclerosis patients (MS) in red, derived using the 2T4k_V_B__k_4_ model, for several grey matter (GM) and white matter (WM) regions of interest. The horizontal blue and red lines represent the mean values for the two subject groups per region. Regions with unreliable parameter estimates (standard deviation for k_3_ > 50%) were excluded. Group differences were analysed using an independent *t* test in SPSS 22.0

V_T_ and BP_ND_ values for the various ROIs are presented in Fig. 3 a and b, respectively. For all regions, mean V_T_ and BP_ND_ were higher for patients than for controls. For V_T_, this group difference was statistically significant (*p* = 0.032–0.048), except for cingulate cortex (*p* = 0.055). It should be noted that when these pilot results were corrected for multiple testing (i.e., after Bonferroni correction *p* < 0.007 would be considered significant); the group differences did not reach statistical significance. For BP_ND_, the group difference did not reach statistical significance for any of the ROIs.

Due to unreliable parameter estimates, as illustrated in Online Resource 2, one healthy control was not included in the thalamic grey matter and two healthy controls were excluded from the hippocampal grey matter. The figures on group differences including these subject are presented in Online Resource 3.

### Multiple sclerosis lesions

**Fig. 4:**
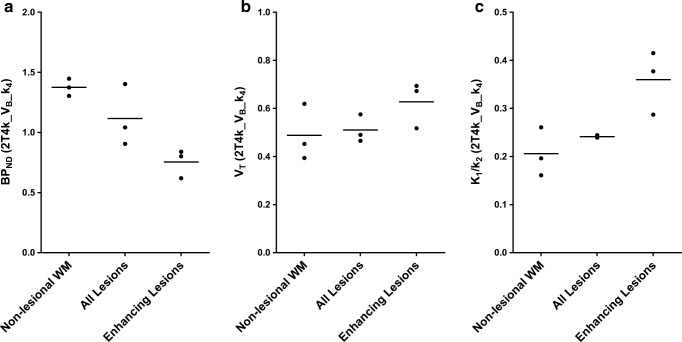
Kinetic parameters for multiple sclerosis lesions. Lesional and non-lesional 2T4k_V_B__k_4_ derived **a** BP_ND_, **b** V_T_, and **c** K_1_/k_2_ for the three relapsing remitting multiple sclerosis patients with analysable gadolinium-enhancing lesions. Mean BP_ND_ was decreased in T2 lesions, and a further decrease was seen in enhancing lesions, whereas for V_T_, an increase was observed. For the K_1_/k_2_ ratio, an even larger increase was observed in the enhancing lesions, most likely due to disruption of the blood-brain barrier in these active lesions

Four patients showed gadolinium-enhancing lesions, but in the patient with the smallest enhancing lesion, the corresponding TAC was too noisy, resulting in unreliable kinetic parameter estimates (subject MS2). Results for the other three subjects are presented in Fig. 4. As Fig. 4a illustrates, mean BP_ND_ decreased in T2 lesions compared with non-lesional white matter. A further decrease was seen in gadolinium-enhancing lesions. In contrast, mean V_T_ was similar for non-lesional white matter and T2 lesions and increased in enhancing lesions (Fig. 4b). In addition, as the blood-brain barrier is disrupted in active MS lesions, the K_1_/k_2_ ratio was, as expected, not constant, also showing an increase in enhancing lesions (Fig. 4c).

### Impact of scan duration

**Fig. 5:**
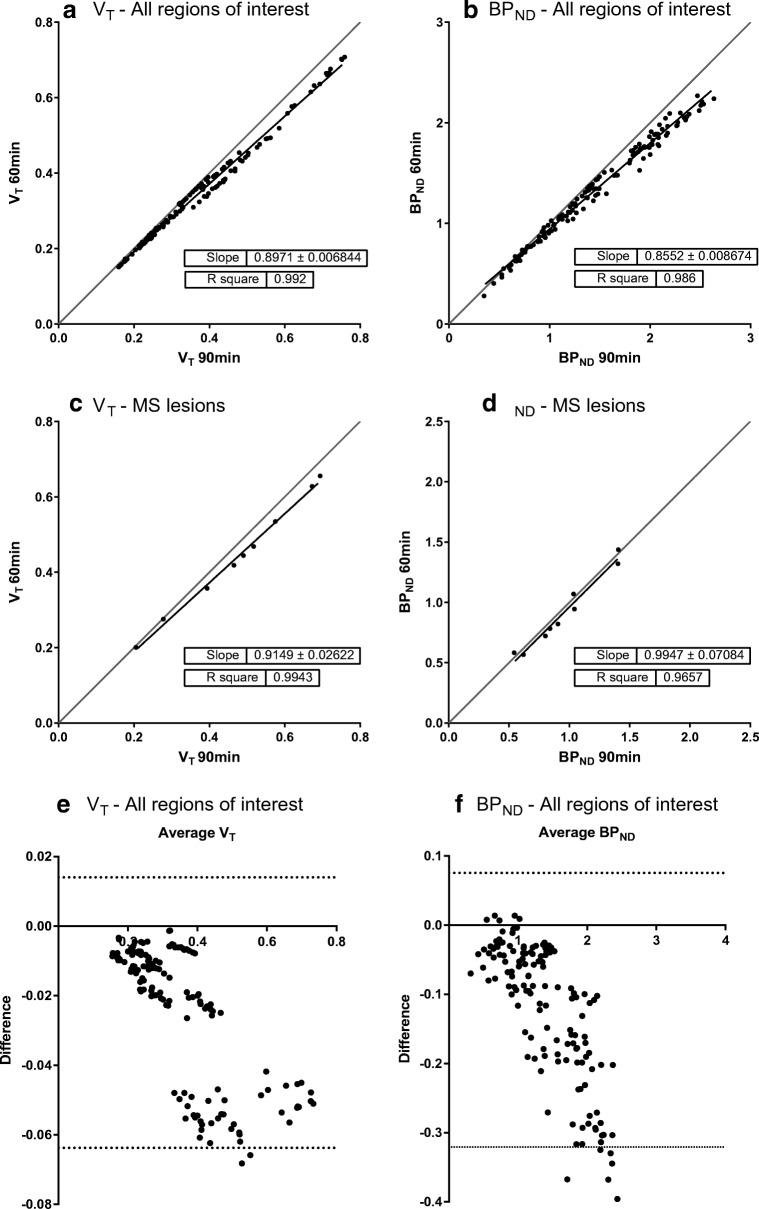
Comparison of the 60-min and 90-min datasets. Linear regression for both **a** V_T_ and **b** BP_ND_ for the combined ROIs showed a very strong correlation between 60- and 90-min datasets. For V_T_, there was a systematic underestimation of approximately 10% when reducing scan duration to 60 min; for BP_ND_ this was about 15%. Linear regression for the T2 lesions and gadolinium-enhancing lesions demonstrated an equally strong correlation for (**c**) V_T_ and (**d**) BP_ND_. Additional Bland-Altman analysis for the combined ROIs (**e**) V_T_ and (**e**) BP_ND_ confirms the results from the linear regression analysis

Regional V_T_ and BP_ND_ values derived from the 60-min data were compared with those from the 90-min data. Figure 5 a shows a very strong correlation for both macro parameters for the combined ROIs, with correlation coefficients (*R*^2^) of 0.992 for V_T_ and 0.986 for BP_ND_. The slopes indicate an underestimation in the values obtained for the 60-min data, approximately 10% for V_T_ and 15% for BP_ND_. Similar results are produces by Bland-Altman analysis. As this underestimation predominantly affected higher parameter values, lower regional group differences were seen for the 60-min data than for the 90 min data, as illustrated in Online Resource 4.

Figure 5 b shows a similar correlation coefficient for V_T_ and BP_ND_ between the two scan durations for the T2 lesions and the gadolinium-enhancing lesions. Just as for the 90-min data, lesions showed lower BP_ND_ than non-lesional brain tissue for the 60-min data as well.

## Discussion

This study shows that binding of [^11^C]SMW139 to the P2X_7_ receptor tracer can be quantified using a two-tissue reversible plasma input model with fixed k_4_. Using this model, it was possible to identify neuroinflammation in both MS lesions and normal appearing brain tissue in RRMS.

In this study, simplified reference tissue models were not evaluated, as the pathophysiology of MS violates the assumptions underlying such models. Firstly, because of global low-grade inflammation in the MS brain, none of the brain regions are devoid of specific binding (P2X_7_ receptors) and hence cannot be considered a reference region [24]. Secondly, due to disruption of the blood-brain barrier in MS, a constant K_1_/k_2_ across the brain cannot be guaranteed [25].

The rate of influx of [^11^C]SMW139 from plasma into the brain was sufficient for accurate data analysis. Regional TACs demonstrated fast efflux of [^11^C]SMW139, which is supported by high k_2_ estimates. Rapidly declining activity in non-displaceable and, consequently, specific compartments was observed, resulting in unreliable estimates especially k_4_ in smaller ROIs. As k_4_ = *k*_off_, it should be constant for the P2X_7_ receptor independent of its location. This limited inter-subject variation for k_4_ for the different ROIs enabled fixing this parameter for smaller regions to the more reliable whole-brain values. By reducing the number of fit parameters, the precision of the other fit parameters was improved, resulting in acceptable SEs for all estimated parameters in most ROIs when using the 2T4k_V_B__k_4_ model.

As demonstrated in Table 2, K_1_ was similar in patient and control groups, but k_2_ was lower in patients, leading to a higher non-displaceable distribution volume (V_ND_) in patients. Nevertheless, apart from V_T_, BP_ND_ (= k_3_/k_4_) was also higher in patients than in controls, suggesting higher specific binding in the patient group than in the controls for both grey and white matter. This implies an increase in activated microglia in normal appearing brain tissue of these clinically active RRMS patients.

The patient who had the lowest BP_ND_ within the patient group for all ROIs had slightly different demographics: longest disease duration (5 years), highest Expanded Disability Status Scale (5.5), slowest timed 25-foot walk (8.0 s), second slowest 9-Hole Peg Test (25.4 s), lowest Symbol Digits Modalities Test (33 correct), and highest T2 lesion load (31.3 cm^2^) with only one small gadolinium-enhancing lesions (0.7 cm^2^). This implies that in this patient MS was more progressed compared with the rest of the group, which in turn could result in less active neuroinflammation and therefore lower P2X_7_ receptor binding [26]. When looking at the demographics of the healthy control with the highest regional BP_ND_ values, this subject’s relatively high age stands out (59.2 years). This could resemble an effect of age on P2X_7_ receptor binding similar to the effect of age on TSPO binding [27], although this clearly needs to be confirmed in a larger cohort.

In the subjects with gadolinium-enhancing lesions, V_T_ was similar for non-lesional white matter and T2 lesions, but increased in the enhancing lesions. In contrast, BP_ND_ values decreased in T2 lesions, with a further decrease in enhancing lesions. As V_T_ includes both non-displaceable and specific compartments (the latter represented by BP_ND_), this indicates that decreased specific binding of [^11^C]SMW139 was compensated by an increased non-displaceable compartment (non-specifically bound or free tracer). This was reflected by an increase in the K_1_/k_2_ ratio in these gadolinium-enhancing lesions. The most likely explanation for this increased K_1_/k_2_ ratio is disruption of the blood-brain barrier in active MS lesions, resulting in increased vascular permeability. Consequently, to quantify [^11^C]SMW139 binding in these active lesions, BP_ND_ is required.

Interestingly, cerebral [^11^C]SMW139 BP_ND_ was increased in RRMS patients compared with healthy controls, but counterintuitively specific binding was decreased in active MS lesions. An explanation for this could be found in the heterogeneity of neuroinflammation in MS. Microglia activation is a complex and dynamic process, and different microglia subtypes and macrophages could be involved in both focal and diffuse neuroinflammation [28].

As carbon-11 has a half-life of 20 min, minimal radioactivity is present in both tissue and blood in the final stage of a 90-min scan. Together with the observed fast kinetics of [^11^C]SMW139, this makes accurate measurements towards the end of the 90-min scans difficult. In addition, a 90-min scan can be quite challenging for both healthy controls and especially patients. Therefore, reducing the scan duration would be beneficial. As shown in Fig. 5, both V_T_ and BP_ND_ obtained from 60-min datasets correlated very strongly with those from 90-min datasets, despite a slight (systematic) underestimation, suggesting that 60-min scans may be sufficient to assess in vivo kinetics of [^11^C]SMW139 without compromising quantitative accuracy (see Online Resource 3). In addition, there appeared to be two clusters in the Bland-Altman analysis of V_T_. These clusters, however, were not present in the Bland-Altman plot of BP_ND_. This suggests that the two clusters may be due to differences in non-specific binding.

As this is a first in-man study, the sample size is rather limited, which is the main limitation of this study. Larger study cohorts are needed to confirm these initial findings. In addition, the small sample size resulted in some demographic differences between patient and control groups, even though they did not reach statistical significance. A possible group difference that could be relevant is the number of smokers, as an effect of smoking on neuroinflammation (in MS) has been reported [29–31]. If and how this could impact *in vivo* [^11^C]SMW139 binding is currently uncertain.

Moreover, a larger sample size would allow for an assessment of possible correlations between [^11^C]SMW139 binding and disease duration, clinical outcome measures such as the EDSS- and MRI-derived outcome measures like atrophy. In addition, [^11^C]SMW139 could potentially be used for longitudinal analyses, which would require additional studies such as test-retest evaluation.

To conclude, this first in-man study demonstrates that a reversible two-tissue plasma input model with fixed k_4_ is the preferred model to quantify [^11^C]SMW139 in healthy controls and RRMS patients.

Additional studies are warranted to further evaluate its clinical relevance as a novel neuroinflammation tracer.

## Electronic supplementary material

Online Resource 1. Additional demographic and PET scanning information. Description: This supplementary file adds to the demographic information provided in Table 1 and provides scanning details per subjects. (PDF 36 kb)

Online Resource 2. Reliability of kinetic parameter estimates for the 2T4k_V_B__k_4_ model. Description: This supplementary file provides the percentage of standard deviation for K_1_, k_2_, k_3_, k_4_ and V_B_ for the large regions of interest. (PDF 106 kb)

Online Resource 3. Regional V_T_ and BP_ND_ values obtained for the full 90 minutes datasets. Description: This supplementary file provides the regional volume of distribution and binding potential values obtained for the full 90 minutes datasets, without exclude regions of interest with unreliable parameter estimates. Group differences were analysed using an Independent T-test in SPSS 22.0. (PNG 65 kb)

High resolution image (EPS 95 kb)

Online Resource 4. Regional V_T_ and BP_ND_ values obtained for 60 minutes datasets. Description: This supplementary file provides the regional volume of distribution and binding potential values obtained for the 60 minutes datasets. Group differences were analysed using an Independent T-test in SPSS 22.0. (PNG 66 kb)

High resolution image (EPS 93 kb)
