# Supplementary material for: The P2X7 receptor tracer [11C]SMW139 as an in vivo marker of neuroinflammation in multiple sclerosis: a first-in man study
Source: Eur J Nucl Med Mol Imaging. 2019 Nov 8;47(2):379–89. doi: 10.1007/s00259-019-04550-x (PMC6974509; doi:10.1007/s00259-019-04550-x)
Supplement: Supplementary file 1 — Additional demographic and PET scanning information. Description: This supplementary file adds to the demographic information provided in Table 1 and provides scanning details per subjects. (PDF 36 kb) [file 259_2019_4550_MOESM1_ESM.pdf]

Online Resource 1. Additional demographic and PET scanning information.

	MS1	MS2	MS3	MS4	MS5	HC1	HC2	HC3	HC4	HC5	*p*-value
**Demographics**											
Age (years)	33	49	51	21	39	31	44	22	27	59	0.84*
Gender (M/F)	F	M	F	F	M	F	M	M	F	F	1.00#
BMI	19	24	22	22	22	20	28	27	27	32	0.20*
Smoking (Y/N)	N	Y	Y	Y	Y	N	N	N	Y	N	0.74#
Disease duration (years)	0.5	5.0	2.5	0.8	0.1						
Expended Disability Status Scale	4.0	5.5	3.0	2.0	2.0						
Other medical history	M. Graves, migraine				Sleep apnea						
Relevant medication	rizatriptan propylthiouracil										
**PET scanning details**											
Time of tracer injection	14:57	11:03	11:08	15:18	14:44	14:17	15:09	15:24	15:14	11:12	0.42*
Injected activity (MBq)	244.0	400.3	344.2	386.5	384.4	360.7	392.2	345.2	378.6	390.0	0.84*
Molar activity (GBq/µmol)	152.3	56.0	17.1	60.5	50.1	98.4	27.3	43.1	63.3	25.2	0.84*

*Mann-Whitney U test, #Chi-square test
