# Supplementary material for: The P2X7 receptor tracer [11C]SMW139 as an in vivo marker of neuroinflammation in multiple sclerosis: a first-in man study
Source: Eur J Nucl Med Mol Imaging. 2019 Nov 8;47(2):379–89. doi: 10.1007/s00259-019-04550-x (PMC6974509; doi:10.1007/s00259-019-04550-x)
Supplement: Supplementary file 2 — Reliability of kinetic parameter estimates for the 2T4k_VB_k4 model. Description: This supplementary file provides the percentage of standard deviation for K1, k2, k3, k4 and VB for the large regions of interest. (PDF 106 kb) [file 259_2019_4550_MOESM2_ESM.pdf]

Online Resource 2. Reliability of kinetic parameter estimates for the 2T4k_V_B_k_4 model

For K_1, k_2 and V_B a cut-off of 25% was used and for k_3 and k_4 a cut-off of 50%. Higher values are highlighted.

A) %SE for K_1

	MS1	MS2	MS3	MS4	MS5	Mean	HC1	HC2	HC3	HC4	HC5	Mean
Frontal cortex	5.5	10.5	12.4	12.6	10.4	10.3	7.6	8.4	10.4	11.9	13.2	10.3
Orbitofrontal cortex	6.6	13.7	14.1	14.1	12.2	12.1	9.0	10.7	10.9	12.3	16.9	12.0
Paracentral cortex	5.8	10.2	11.4	11.0	9.9	9.7	7.4	8.4	9.8	10.8	12.0	9.7
Parietal cortex	6.4	8.7	10.6	10.0	8.9	8.9	6.4	7.6	8.2	10.8	10.9	8.8
Temporal cortex	5.1	9.2	11.9	10.6	8.6	9.1	6.2	8.9	8.4	11.6	10.7	9.2
Occipital cortex	4.6	8.8	8.8	7.2	5.9	7.1	4.1	7.1	6.6	8.4	6.8	6.6
Cingulate cortex	9.9	15.2	12.9	15.6	13.8	13.5	10.2	11.2	13.1	13.9	17.5	13.2
Thalamic GM	10.4	10.8	10.1	8.8	10.6	10.1	3.8	7.1	14.1	11.4	11.2	9.5
Putaminal GM	5.3	12.7	13.3	11.2	12.4	11.0	6.4	14.3	10.8	19.2	12.5	12.6
Caudate nucleus GM	6.2	10.9	13.0	13.8	11.0	11.0	9.2	15.0	15.7	12.2	13.3	13.1
Hippocampal GM	8.3	13.9	15.0	18.5	14.3	14.0	12.0	19.2	12.5	20.3	16.7	16.1
Cerebellar GM	3.6	5.6	7.6	5.7	5.6	5.6	5.8	5.1	5.2	8.8	6.1	6.2
Cerebellar WM	6.0	5.2	6.7	6.1	6.2	6.0	7.1	6.5	7.1	11.1	8.0	8.0
Brainstem WM	8.1	7.9	10.3	8.2	14.3	9.8	15.1	9.8	9.8	13.1	9.5	11.5
Non-lesional WM	6.6	9.6	8.7	8.0	8.6	8.3	8.4	9.1	9.5	11.5	10.6	9.8
T2 MS lesions	6.4	11.0	4.8	5.5	7.7	7.1						
Enhancing MS lesions	8.0	19.4	4.5	4.2		9.0						

B) %SE for K_2

	MS1	MS2	MS3	MS4	MS5	Mean	HC1	HC2	HC3	HC4	HC5	Mean
Frontal cortex	6.9	13.4	15.9	15.6	13.8	13.1	9.9	9.5	12.9	13.9	15.0	12.2
Orbitofrontal cortex	8.3	18.2	18.4	17.5	16.3	15.7	11.6	12.4	13.4	14.2	19.2	14.2
Paracentral cortex	7.4	13.3	14.9	13.9	13.2	12.5	9.7	9.7	12.1	12.8	13.7	11.6
Parietal cortex	8.0	11.3	13.8	12.5	11.7	11.5	8.2	8.8	10.2	12.5	12.2	10.4
Temporal cortex	6.5	12.0	15.6	13.4	11.5	11.8	8.3	10.5	10.7	13.7	12.3	11.1
Occipital cortex	5.7	11.2	11.5	9.0	7.8	9.0	5.2	8.3	8.3	9.8	7.7	7.9
Cingulate cortex	12.8	19.5	17.0	19.8	18.6	17.5	13.7	13.1	16.2	16.4	20.8	16.0
Thalamic GM	12.7	12.8	12.6	10.4	13.4	12.4	4.7	8.0	17.0	13.2	12.3	11.0
Putaminal GM	6.6	15.6	16.0	13.2	15.6	13.4	8.2	15.5	13.1	21.6	13.3	14.3
Caudate nucleus GM	7.4	13.1	15.4	16.9	14.1	13.4	11.0	16.0	18.7	14.1	14.7	14.9
Hippocampal GM	10.7	17.6	19.7	24.6	19.6	18.4	16.5	23.5	16.7	25.4	19.7	20.4
Cerebellar GM	4.5	6.9	9.4	6.9	7.2	7.0	7.1	5.6	6.4	9.8	6.6	7.1
Cerebellar WM	9.3	8.2	10.4	9.1	9.8	9.4	11.7	9.4	11.2	16.3	10.8	11.9
Brainstem WM	11.9	12.1	15.1	11.9	22.0	14.6	25.1	13.3	15.3	19.0	12.7	17.1
Non-lesional WM	10.7	14.9	14.4	12.6	14.8	13.5	14.4	13.5	15.6	17.8	14.8	15.2
T2 MS lesions	10.8	20.8	9.4	9.7	14.9	13.1						
Enhancing MS lesions	14.3	39.2	9.0	8.5		17.8						

C) %SE for K_3

	MS1	MS2	MS3	MS4	MS5	Mean	HC1	HC2	HC3	HC4	HC5	Mean
Frontal cortex	13.2	22.3	20.4	21.2	17.2	18.9	17.1	20.5	21.4	29.8	21.1	22.0
Orbitofrontal cortex	15.0	33.7	24.6	21.6	22.6	23.5	19.8	27.1	23.4	31.0	27.4	25.7
Paracentral cortex	14.2	19.1	19.2	19.2	15.9	17.5	16.0	18.1	18.9	27.9	19.0	20.0
Parietal cortex	14.9	16.6	17.1	16.0	14.2	15.8	13.1	15.7	15.7	25.8	15.7	17.2
Temporal cortex	12.6	21.8	21.0	19.1	16.3	18.2	15.6	21.7	19.6	31.0	17.5	21.1
Occipital cortex	11.0	16.4	14.4	11.6	10.0	12.7	8.0	12.4	12.6	19.9	9.4	12.5
Cingulate cortex	24.1	35.9	23.6	31.3	26.1	28.2	23.8	29.9	30.4	40.7	31.4	31.2
Thalamic GM	31.7	25.9	18.6	19.5	23.4	23.8	13.9	25.6	39.9	53.9	22.8	31.2
Putaminal GM	18.0	36.7	22.2	20.5	26.1	24.7	23.6	43.2	33.7	68.1	24.7	38.7
Caudate nucleus GM	15.1	24.4	17.7	25.9	18.9	20.4	20.5	32.5	32.1	33.5	19.6	27.6
Hippocampal GM	19.5	38.4	27.3	38.1	30.6	30.8	35.8	55.5	36.4	74.6	34.2	47.3
Cerebellar GM	9.3	13.1	11.1	9.3	9.2	10.4	11.6	10.8	12.4	26.5	9.3	14.1
Cerebellar WM	18.7	15.6	18.9	18.0	17.2	17.7	22.8	21.2	19.8	35.0	17.6	23.3
Brainstem WM	23.5	27.2	25.3	20.9	35.2	26.4	51.5	29.1	29.1	48.0	21.1	35.8
Non-lesional WM	18.7	22.6	25.4	24.7	23.2	22.9	21.1	25.5	22.7	30.5	21.9	24.3
T2 MS lesions	18.9	38.5	26.4	24.0	24.1	26.4						
Enhancing MS lesions	39.3	128.3	28.5	35.7		58.0						

D) %SE for V_B

	MS1	MS2	MS3	MS4	MS5	Mean	HC1	HC2	HC3	HC4	HC5	Mean
Frontal cortex	6.5	9.9	10.5	8.9	9.5	9.1	5.1	6.2	8.0	8.3	8.5	7.2
Orbitofrontal cortex	7.9	14.7	11.9	11.6	11.4	11.5	6.6	8.1	9.1	9.6	11.8	9.0
Paracentral cortex	7.2	9.1	9.7	8.1	9.0	8.6	5.2	6.1	8.0	7.6	7.8	6.9
Parietal cortex	7.5	8.7	8.8	7.4	7.7	8.0	4.6	5.3	6.3	6.8	6.9	6.0
Temporal cortex	7.0	9.5	10.7	8.3	8.0	8.7	4.6	6.4	6.9	8.5	7.7	6.8
Occipital cortex	5.0	8.4	6.1	4.4	4.2	5.6	2.6	4.2	4.5	5.0	4.0	4.1
Cingulate cortex	12.1	13.0	10.8	11.7	12.1	11.9	6.3	7.6	10.5	8.4	11.8	8.9
Thalamic GM	13.7	9.9	7.4	8.8	10.4	10.0	3.4	5.5	13.8	8.9	7.5	7.8
Putaminal GM	7.6	13.0	11.6	9.5	12.5	10.8	5.4	11.7	12.5	15.2	10.4	11.0
Caudate nucleus GM	8.5	12.4	11.5	11.2	9.6	10.6	7.8	14.1	16.8	10.4	8.7	11.6
Hippocampal GM	10.7	14.9	15.6	13.0	15.0	13.8	10.0	13.9	11.8	12.6	11.4	11.9
Cerebellar GM	5.0	6.3	5.9	4.4	4.7	5.3	4.4	3.8	4.4	5.3	4.7	4.5
Cerebellar WM	8.6	6.9	8.2	6.9	6.8	7.5	6.5	5.7	7.4	8.2	7.3	7.0
Brainstem WM	14.5	11.4	11.2	9.8	14.5	12.3	11.9	9.4	10.8	10.2	8.8	10.2
Non-lesional WM	10.2	10.6	10.8	9.2	10.0	10.2	6.7	8.2	9.2	9.2	8.8	8.4
T2 MS lesions	11.2	15.2	10.2	8.5	8.6	10.7						
Enhancing MS lesions	17.7	26.1	10.3	12.6		16.7						

Abbreviations: GM = grey matter, HC = healthy control, MS = multiple sclerosis patient, SE = standard error, WM = white matter
