# Supplementary figures and images for: The P2X7 receptor tracer [11C]SMW139 as an in vivo marker of neuroinflammation in multiple sclerosis: a first-in man study
Source: Eur J Nucl Med Mol Imaging. 2019 Nov 8;47(2):379–89. doi: 10.1007/s00259-019-04550-x (PMC6974509; doi:10.1007/s00259-019-04550-x)

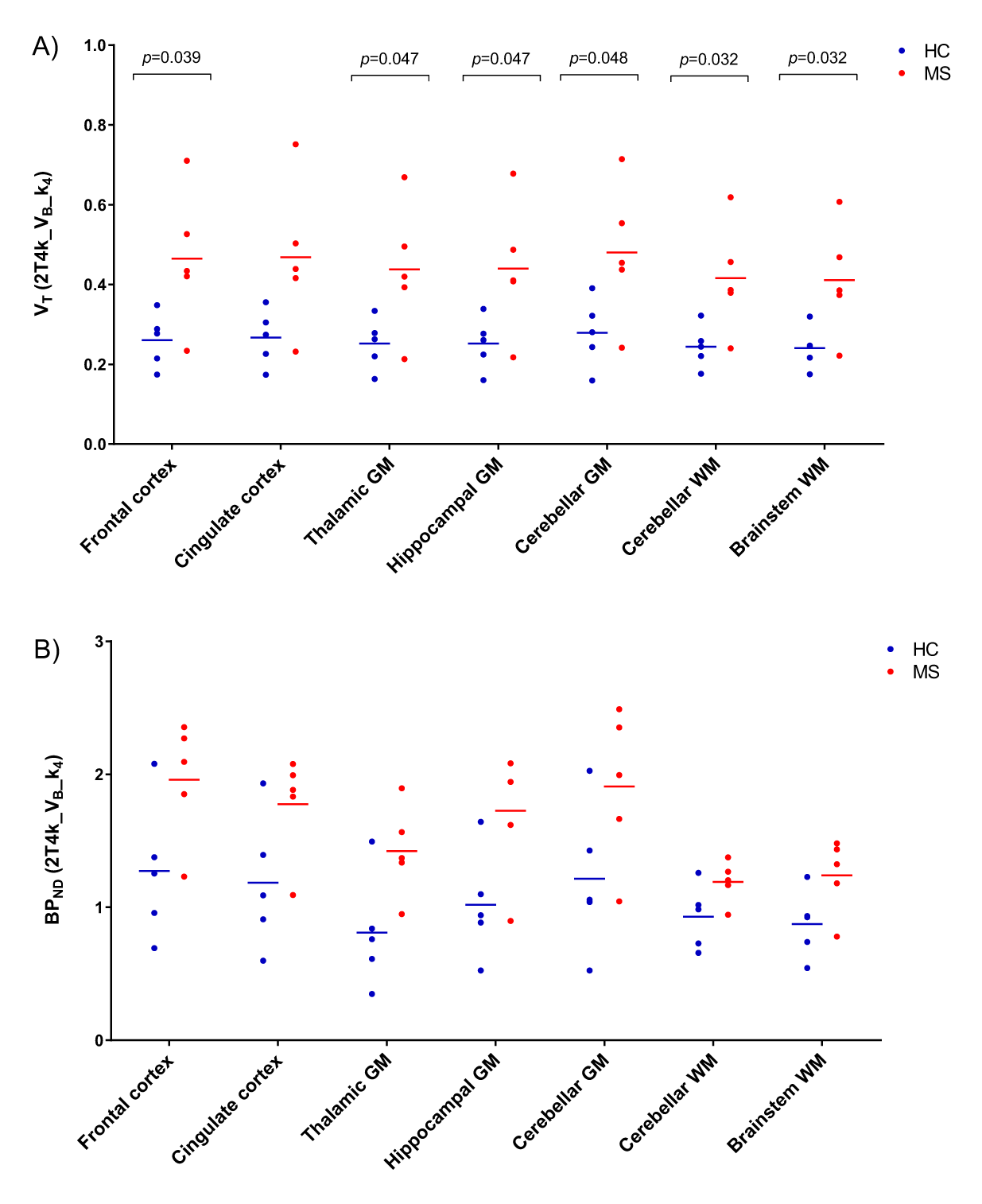

Supplement: Supplementary file 3 — Regional VT and BPND values obtained for the full 90 minutes datasets. Description: This supplementary file provides the regional volume of distribution and binding potential values obtained for the full 90 minutes datasets, without exclude regions of interest with unreliable parameter estimates. Group differences were analysed using an Independent T-test in SPSS 22.0. (PNG 65 kb) [file 259_2019_4550_Fig6_ESM.png]

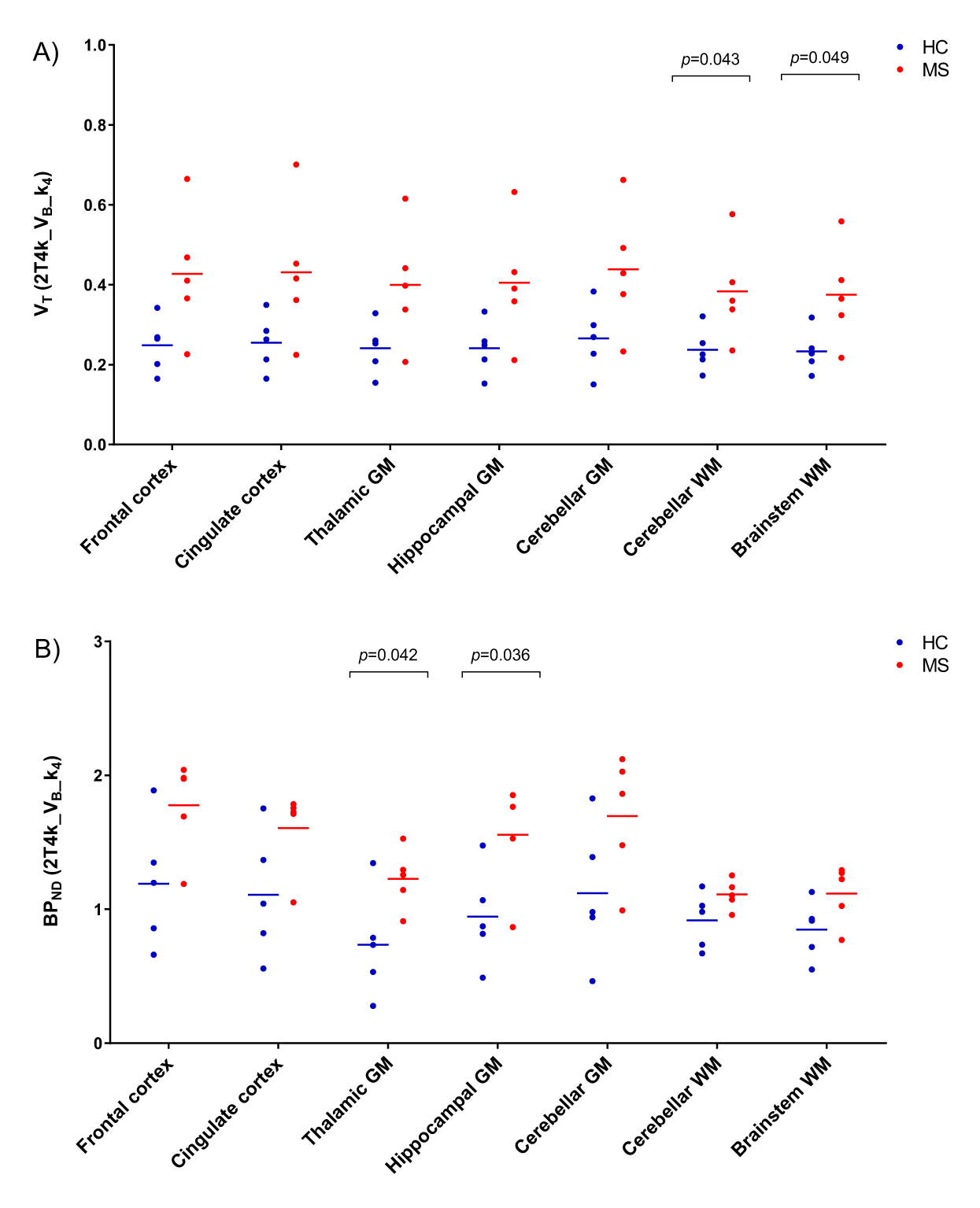

Supplement: Supplementary file 5 — Regional VT and BPND values obtained for 60 minutes datasets. Description: This supplementary file provides the regional volume of distribution and binding potential values obtained for the 60 minutes datasets. Group differences were analysed using an Independent T-test in SPSS 22.0. (PNG 66 kb) [file 259_2019_4550_Fig7_ESM.png]
